# Prevalence and Seroincidence of Hepatitis B and Hepatitis C Infection in High Risk People Who Inject Drugs in China and Thailand

**DOI:** 10.1155/2014/296958

**Published:** 2014-03-27

**Authors:** J. Brooks Jackson, Liu Wei, Fu Liping, Apinun Aramrattana, David D. Celentano, Louise Walshe, Yi Xing, Paul Richardson, Ma Jun, Geetha Beauchamp, Deborah Donnell, Yuhua Ruan, Liying Ma, David Metzger, Yiming Shao

**Affiliations:** ^1^Department of Pathology, Johns Hopkins University, Baltimore, MD 21287, USA; ^2^Guangxi Center for Disease Control and Prevention, Nanning 530028, China; ^3^Xinjiang Center for Disease Control and Prevention, Urumqi 83001, China; ^4^Research Institute for Health Sciences, Chiang Mai University, Chiang Mai 50200, Thailand; ^5^Department of Epidemiology, Johns Hopkins Bloomberg School of Public Health, Baltimore, MD 21205, USA; ^6^Fred Hutchinson Cancer Research Center, Seattle, WA 98109, USA; ^7^State Key Laboratory for Infectious Disease Prevention and Control, National Center for AIDS/STD Control and Prevention, Chinese Center for Disease Control and Prevention, Collaborative Innovation Center for Diagnosis and Treatment of Infectious Diseases, Beijing 102206, China; ^8^University of Pennsylvania, Philadelphia, PA 19104, USA

## Abstract

We determined the prevalence and incidence of HBV and HCV infection in people who inject drugs (PWIDs) at high risk for HIV in China and Thailand and determined the association of HBV and HCV incidence with urine opiate test results and with short-term versus long-term buprenorphine-naloxone (B-N) treatment use in a randomized clinical trial (HPTN 058). 13.8% of 1049 PWIDs in China and 13.9% of 201 PWIDs in Thailand were HBsAg positive at baseline. Among HBsAg negative participants, the HBsAg incidence rate was 2.7/100 person years in China and 0/100 person years in Thailand. 81.9% of 1049 PWIDs in China and 59.7% of 201 in Thailand were HCV antibody positive at baseline. The HCV confirmed seroincidence rate among HCV antibody negative PWIDs was 22/100 person years in China and 4.6/100 person years in Thailand. Incident HBsAg was not significantly different in the short-term versus long-term B-N arm in China or Thailand. Participants with positive opiate results in at least 75% of their urines during the time period were at increased risk of incident HBsAg (HR = 5.22; 95% CI, 1.08 to 25.22; *P* = 0.04) in China, but not incident HCV conversion in China or Thailand.

## 1. Background and Objectives

Transmission of hepatitis C virus (HCV), hepatitis B virus (HBV), and human immunodeficiency virus (HIV-1) has been causally associated with the injection of drugs of abuse by people who inject drugs (PWIDs) due to sharing of needles and injection equipment contaminated by infected blood. HCV and HBV seroprevalence rates among PWIDs have varied considerably depending on the geographic region and time period of the PWID populations tested.

HCV seroprevalence rates among most PWIDs populations worldwide have been reported to be often higher than 50% with widely varying rates of 3–95% for HIV/HCV coinfection [1]. In China, HCV prevalence rates among the general population have been estimated to be approximately 3.2% [2] with HCV prevalence rates of between 34% and 99% among PWID populations [2]. In Thailand, an HCV prevalence rate of 70% among PWIDs has been reported [3]. HCV coinfection complicates HIV treatment options and has been reported to increase the probability of progression to a new AIDS-defining clinical event or to death and is associated with a smaller CD4-cell recovery increase in response to HIV therapy [4].

Hepatitis B surface antigen (HBsAg) prevalence rates in China have been relatively high with an estimated 7.18% rate in the general population in 2006 [5]. In Thailand, the HBsAg prevalence rate among HIV infected Thai patients has been reported to be 8.7% [6]. Like HCV coinfection, HBV coinfection is associated with increased risk of HIV progression and death [7] and also complicates HIV treatment options as several antiretroviral drugs have activity against both HIV and HBV.

Between 2007 and 2011 we conducted an open-label randomized clinical trial of buprenorphine-naloxone (B-N) treatment of opiate dependence as a strategy for prevention of HIV in opiate dependent injectors in Chiang Mai, Thailand; Heng County and Nanning city, Guangxi, China; and Urumqi, Xinjiang, China. All PWIDs enrolled were HIV negative adults and had injected opiates at least 12 times in the last 28 days and had a positive urine test for opiates. Approximately 44%, 14%, 7%, and 53% of PWIDs in the study reported sharing needles or works during the 6 months prior to enrollment in Heng County, Nanning, Urumqi, and Chiang Mai, respectively.

Participants were randomized in a 1 : 1 ratio to a short-term arm consisting of detoxification using B-N combined with 12 months of behavioral and drug and risk counseling (BDRC) or to a long-term arm consisting of taking B-N three times per week and BDRC for 12 months. Rates of HIV-infection (determined at screening, and at 26 and 52 weeks of follow-up) and death were compared one year after completing the interventions. During the first year, 39% of monthly visits in the long-term arm versus 69% in the short-term arm had positive urine drug screens (OR = 0.3, *P* < 0.001) [8]. The objective of this substudy was to determine the seroprevalence and incidence of HCV antibody and HBsAg among these initially HIV negative PWIDs and to determine the association of HBV and HCV incidence with urine opiate test results and with short-term versus long-term B-N treatment use at follow-up.

At the time of enrolment, participants in China gave consent for storage of specimens for future testing, whereas participants in Thailand were not asked and only protocol testing was performed. Approvals for HCV and HBV testing of these specimens among these initially HIV negative PWIDs were obtained from the local Institutional Review Boards in China, Thailand, and Johns Hopkins University in the United States. In Thailand, participants found to be negative for HBsAg and HBsAb at baseline were offered HBV vaccination, whereas in China, all participants found to be HBsAg negative were offered HBV vaccine, so some may have been HBsAb positive.

## 2. Methods

Serum samples from 1049 HIV antibody negative PWIDs in Heng County, Nanning, and Urumqi, China (963 men and 86 women) were tested at baseline and between 26 and 52 weeks later for HBsAg using a commercial enzyme immunoassay (EIA) (Abbott Murex HBsAg version 3.0) and for HCV antibody using two different HCV EIA assays (Ortho HCV antibody version 3.0 and Wantai HCV antibody assay) at baseline and between 26 and 156 weeks later. If the HBsAg test was initially nonreactive, then the participant was considered to be negative for HBsAg. If the HBsAg test was initially reactive, then it was repeated in duplicate. If at least two of 3 tests were reactive, then the participant was considered to be positive for HBsAg. For HCV testing, if both HCV EIA antibody assays were nonreactive, then the participant was considered not to be HCV infected. If either assay was reactive, then the Ortho HCV assay was repeated in duplicate. If two of 3 Ortho HCV assays were reactive, then the participant was considered to be HCV infected. Samples that were repeatedly reactive for HCV antibody at a follow-up visit were tested for HCV RNA by the Roche COBAS AmpliPrep/COBAS TaqMan HCV assay. Not all participants had follow-up testing performed in China due to early closure of the study by the Data Safety Monitoring Board on account of futility due to a low HIV incidence (the primary study endpoint).

In Thailand, serum samples from approximately 201 HIV antibody negative PWIDs (188 men and 13 women) were tested at baseline for HBsAb and HBsAg using the AxSYM assay (Abbott Laboratories). Seventy-three of 201 participants who were found to be HBsAg and HBsAb negative were offered HBV vaccine of whom 68 received all 3 HBV vaccinations. They were tested at 26 and 52 weeks after enrollment for HBsAg using a commercial assay (AxSYM version 2, Abbott Laboratories) and all 201 participants were tested for HCV antibody (AxSYM HCV test Version 3, Abbott Laboratories). If the HBsAg test was initially nonreactive, then the participant was considered to be negative for HBsAg. If the HBsAg test was initially reactive, then it was repeated in duplicate. If at least two of 3 tests were reactive, then the participant was considered to be positive for HBsAg. For HCV testing, if the HCV EIA antibody AxSYM assay was initially nonreactive, then the participant was considered not to be HCV infected. If the assay was reactive, then the sample was retested using another assay (Murex anti HCV version 4.0, Abbott Laboratories). If both tests were reactive, the individual was considered HCV antibody positive. If the Murex HCV antibody test was negative, then the result was considered indeterminate.

Urine tests for opiate use were performed every 4 weeks during the first 52 weeks and then semi-annually (weeks 78, 104, 130, and 156). In all 4 sites, the same commercial test for detection of urine opiates with a sensitivity (cut off) of 300 ng/mL was used according to the manufacturer's directions (Integrated E-Z Split Key Cup, Acon Laboratories, San Diego, CA).

### 2.1. Statistical Analysis

Seroincidence rates and confidence intervals were calculated based on a Poisson distribution, with time of infection set to time of first antibody positive test. Cox proportional hazards models were used to compute hazard ratios using time to first positive test among those negative at baseline, censoring participants at their final test visit for HBV and HCV, respectively. Associations with positive urine opiate tests were assessed using the proportion of positive opiate urine tests calculated over each 26 week testing interval for each participant. All analyses were conducted using SAS 9.2 (SAS, Inc).

## 3. Results

As shown in Table 1, 145 (13.8%) of 1049 PWIDs in China were HBsAg positive at baseline [80 (19.5%) of 411 PWIDs in Heng County, Guangxi, 16 (9.9%) of 161 in Nanning Guangxi, and 49 (10.3%) of 477 in Urumqi, Xinjiang]. Of the 904 HBsAg negative participants at baseline, 607 (67%) were tested at follow-up; 9 had detectable HBsAg, demonstrating an HBsAg incidence rate of 2.7/100 person years. The demographic data (age, gender, marital status) for HBsAg negative participants by country at baseline are shown in Table 2.

**Figure 1:**
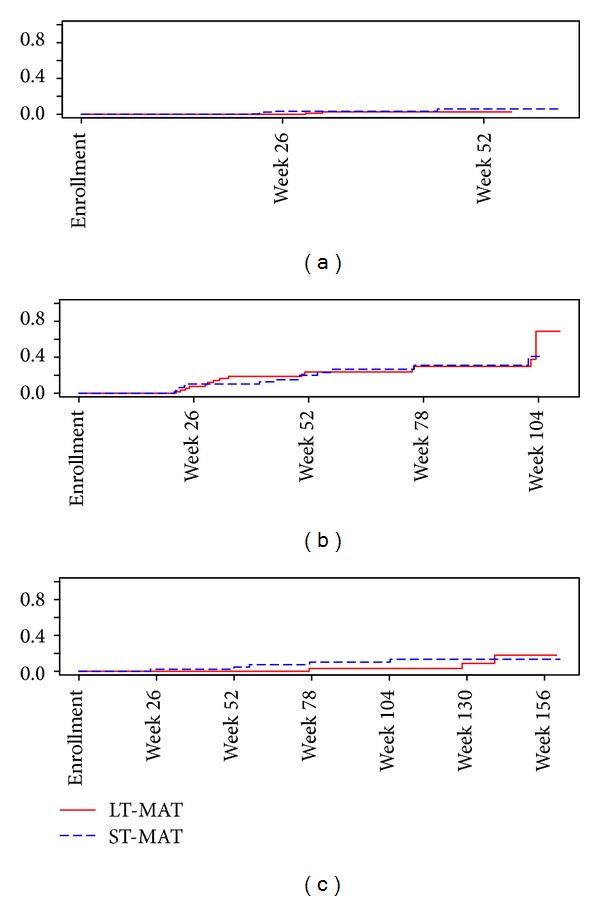
(a) Cumulative probability of Hepatitis B in China. (b) Cumulative probability of Hepatitis C in China. (c) Cumulative Probability of Hepatitis C in Thailand.

In PWIDs in China, the incidence of becoming HBsAg positive in the short- versus long-term B-N treatment was 4.34/100 person years versus 1.15/100 person years (HR = 3.55; 95% CI 0.74 to 17.08, *P* = 0.11) with the Kaplan Meier curve shown by treatment arm in Figure 1(a). In the first 26 weeks of the study, across both arms of the study 40% of the initially HBsAg negative participants in China had at least 75% of their urine samples test positive for opiates and 44% between 26 and 52 weeks. Those with positive urine opiate results in at least 75% of their urines during the time period were at increased risk of incident HBsAg (HR = 5.22; 95% CI, 1.08 to 25.22; *P* = 0.04).

In terms of HCV antibody, 859 (81.9%) of 1049 PWIDs in China were HCV antibody positive at baseline [322 (78.4%) of 411 PWIDs in Heng County, Guangxi; 150 (93.2%) of 161 in Nanning, Guangxi; and 387 (81.1%) of 477 in Urumqi, Xinjiang]. The percentage of women and men who were HCV antibody positive at baseline was 94% and 81%, respectively. The percentage of those who were HCV antibody positive at baseline of less than or equal to 34 years of age compared with more than 34 years of age was 77% and 88%, respectively. Of the 190 HCV antibody negative participants, 132 had follow-up samples at 26–156 weeks of which 41 had detectable HCV antibody by repeating EIA testing). The demographic data (age, gender, and marital status) for HCV antibody negative participants by country at baseline are shown in Table 3. HCV RNA PCR testing confirmed HCV infection in 29 of 41 antibody repeatedly reactive samples yielding an overall confirmed seroincidence rate of 22/100 person years with the Kaplan Meier curve shown in Figure 1(b). Heng County, Guangxi, had an incidence rate of 20.9 per 100 years [95% CI, 10.8 to 36.4]; Nanning, Guangxi, 27.7 per 100 years [95% CI, 0.7 to 154]; and Urumqi, Xinjiang, 22.6 per 100 person years [95% CI, 12.9 to 36.7].

Overall, in China, short-term versus long-term B-N treatment was not associated with HCV infection (HR = 0.95; 95% CI, 0.45 to 1.97; *P* = 0.9) with the Kaplan Meier curve shown in Figure 1 by treatment arm. In the first 26 weeks of the study, 42% of the initially HCV uninfected participants in China had at least 75% of their urine test positive for opiates; between 26 and 52 weeks this decreased to 34%, and post intervention increased to 62% at week 78 and 58% at week 104. Those with positive opiate results in at least 75% of their urines during the time period did not appear at increased risk of incident HCV infection (HR = 1.044; 95% CI, 0.48 to 2.23; *P* = 0.91).

In Thailand, 28 (13.9%) of 201 PWIDs were HBsAg positive at baseline. Of the 173 HBsAg and HBsAb negative participants, 55 (31.8%) had follow-up samples six months later of which none had detectable HBsAg or 0/100 person years. In terms of HCV, 120 (59.7%) of 201 PWIDs were HCV antibody positive at baseline in Thailand. The percentage of women and men who were HCV antibody positive at baseline was 46% and 61%, respectively. The percentage of those who were HCV antibody positive at baseline of less than or equal to 34 years of age compared with more than 34 years of age was 65% and 56%, respectively. Of the 81 HCV antibody negative participants, 78 had follow-up samples of which 8 had detectable HCV antibody for an incidence rate of 4.59 per 100 person years [95% CI, 2.0 to 9.0]. Short-term versus long-term B-N treatment was not associated with HCV infection in Thailand (HR = 1.39; 95% CI, 0.33 to 5.83, *P* = 0.65) with the Kaplan Meier curve shown by treatment arm in Figure 1(c). Those with positive opiate results in at least 75% of their urines during the time period did not appear at increased risk of incident HCV infection (HR = 2.85; 95% CI, 0.55 to 14.58; *P* = 0.21).

Among the HIV antibody negative participants who were either HCV uninfected or HBsAg negative at baseline, there were no HIV incident cases in Thailand (0/55) and 6 incident HIV cases in China among the 607 HBsAg negative participants in follow-up for an HIV incidence rate of 0.81/100 person years (interquartile range 0.30, 1.76). These 6 cases were among 586 participants who remained HBsAg negative in follow-up.

There was one HIV incident case among 132 participants in follow-up in China who was HCV uninfected at baseline for an incidence rate of 0.66/100 person years (interquartile range (0.02, 3.70). This one HIV incident case was among 29 subjects who became HCV infected during follow-up.

## 4. Discussion

The prevalence and incidence of HBsAg and HCV infection among HIV negative PWIDs in 3 sites in China and one site in Thailand were very high. The baseline HBsAg prevalence in China and Thailand was nearly identical at 13.8% and 13.9%, respectively, nearly twice as high as that reported in the general population [5]. HBV vaccine and HBV immunoglobulin to prevent perinatal transmission have been available for a couple of decades. However, the mean age and range of Thai participants who were HbSAg positive at baseline were 37 and 23 to 48 years, respectively, and for Chinese participants who were HbSAg positive at baseline were 32 and 18 to 49 years, respectively. Given this older cohort, it is likely they did not receive HBV vaccination perinatally as newborns. This difference probably reflects the additional risk of injection use above that caused by perinatal transmission in these populations. However, the relatively low HBsAg incidence rates of 2.7/100 person years in China and 0/100 persons years in Thailand are not surprising given the high background rates of HBV infection and clearance in high hepatitis endemic areas [9] and the fact that a number of HBsAg negative participants at baseline agreed to the receipt of HBV vaccine. For example, in Thailand, all HBsAg negative and HBsAb negative participants received HBV vaccine, which probably explains the zero incidence of HBV infection, whereas in China at least 29% (260 of 940) HBsAg negative PWIDs at baseline did not receive HBV vaccine. Given that HBsAb was not measured in the Chinese participants at any time in the study, some HBsAg negative participants at baseline may have been immune at baseline and/or after vaccination. However, there appear to be a number of susceptibles who became HBsAg positive either because they did not receive the vaccine or were given the vaccine, but they became infected shortly after enrollment as all of the incident cases occurred by week 26.

In terms of hepatitis C, approximately 82% and 60% of PWIDs in this study in China and Thailand, respectively, were HCV antibody positive at baseline which is consistent with reported prevalence rates in these countries [1, 2]. The HCV incidence rates were 22/100 person years in China compared with 4.6/100 person years in Thailand which may reflect the higher prevalence rate at baseline or higher risk injection practices associated with increased sharing and/or higher frequency of injection in China.

Those participants with positive opiate urine tests in at least 75% of the urines over the study period were at increased risk of incident HBsAg conversion, but they were not at increased risk for HCV antibody conversion. HBsAg and HCV antibody incidence rates were not significantly associated with short-term versus long-term B-N treatment arms, although in China, the HBsAg incidence rate was more than 3-fold higher in the short-term B-N arm and nearly 5-fold higher in those participants with positive opiate results in at least 75% of their urines at follow-up.

Why there was not a significantly higher HCV incidence associated with positive urine opiate tests is not clear. Perhaps the number of susceptible participants in this high HCV prevalence population was too small to discern a difference, or the efficiency of HCV transmission is so high through needle sharing that differences in incident infection will not be significant given that 39% of monthly visits in the long term B-N arm had positive urine drug screens. Another possible explanation is that many of the Thai participants were mostly from ethnic minorities whose networks appeared small and closed. Perhaps HCV had not reached some of these closed networks. Many of the PWIDs also smoked opium or may have injected opium less frequently which would give positive urine results, yet be associated with a lower risk of HCV transmission.

These findings support the concept that decreased injection drug use, as evidenced by negative urine opiate tests associated with the long term B-N arm, will likely lead to decreased HBsAg incidence rates.

## Figures and Tables

**Table 1:** HBV and HCV prevalence and incidence rates for HBV and HCV at 3 sites in China and one site in Thailand; (95% confidence intervals).

	3 sites in China	Chiang Mai, Thailand
HBsAg prevalence at baseline	13.8% (11.7, 15.9)	13.9% (9.1, 18.7)
HCV Ab prevalence at baseline	81.9% (80.7, 83.1)	59.7% (56.2, 63.2)
HBsAg incidence	2.7/100 py (1.2, 5.1)	0/100 py (0.00, 10.1)
HCV Ab incidence	22/100 py (14.7, 31.6)	4.6/100 py (1.9, 9.0)

**Table 2:** Demographics data for HBsAg negative participants at 3 sites in China and one site in Thailand.

	3 sites in China *N* = 607	Chiang Mai, Thailand *N* = 55
Age (mean, range)	33 (18–54)	37 (18–65)
Males	92% (556)	96% (53)
Married/living with partner	48% (291)	78% (43)

**Table 3:** Demographics data for HCV uninfected participants negative at 3 sites in China and one site in Thailand.

	3 sites in China *N* = 132	Chiang Mai, Thailand *N* = 78
Age (mean, range)	30 (18–54)	38 (19–65)
Males	96% (127)	91% (71)
Married/living with partner	52% (68)	76% (59)
